# Antimicrobial resistance in Brazil: an integrated research agenda

**DOI:** 10.1590/1980-220X-REEUSP-2021-0589

**Published:** 2022-02-16

**Authors:** Juliana Silva Corrêa, Luiz Felipe Zago, Roberto Rubem da Silva-Brandão, Sandi Michele de Oliveira, Lislaine Aparecida Fracolli, Maria Clara Padoveze, Gloria Cristina Cordoba Currea

**Affiliations:** 1 Universidade de São Paulo, Escola de Enfermagem, São Paulo, SP, Brazil.; 2 Universidade de São Paulo, Faculdade de Saúde Pública, São Paulo, SP, Brazil.; 3 University of Copenhagen, Institute of Public Health, Copenhagen, Denmark.

## INTRODUCTION

Acquired antimicrobial resistance (AMR) occurs when bacteria, viruses, fungi, and parasites change over time and cease to respond to drugs to which they were previously ­susceptible^(1)^. This makes infections more difficult to treat and increases the risk of pathogens spreading, leading to higher mortality. Although the term antimicrobial refers to antibiotics, antivirals, antifungals, and antiparasitics, this article focuses on issues related to the use of antibiotics in the Brazilian context.

AMR is a threat to global health and its worsening is observed in the current context of the SARS-CoV-2 pandemic. Added to the challenges of infection control and the prevalence of morbidity and mortality in the pandemic, viral and bacterial resistance are a global health emergency. In this regard, AMR becomes intrinsically related to the current health context, which may imply accelerated AMR patterns and greater incidence in health services.

These challenges, with health and political implications on a global scale, are especially difficult in countries with medium and low economic income, as is the case of Latin America. In this scenario, responses to AMR require coordinated and ­multisectoral efforts involving clinical-biological, socio­economic, and political ­perspectives at the global, national, and local levels.

In response to the global agenda for tackling AMR, the World Health Organization (WHO) proposes “a collaborative, multisectoral, and transdisciplinary approach – working at local, regional, and national levels – with the goal of achieving optimal health and well-being outcomes, recognizing the interconnection between people, animals, plants, and their shared environment”, designated One Health^(2,3)^.

This article considers the potential ramifications of the One Health approach to AMR research. It adopts a theoretical-methodological perspective that articulates the One Health approach and the social production of the AMR phenomenon, in three axes. The first refers to the practices of prescribing and dispensing antibiotics by professionals. The second one concerns the analysis of antibiotic use from the point of view of health services users. The third one proposes a reflection on the process of construction of a political agenda to fight AMR in Brazil. This contribution presents an analysis and appreciation of the diversity of practices, knowledge, and individual perspectives of Primary Health Care (PHC) users, health professionals, and policy makers that aim to fill research and knowledge gaps in the Brazilian context. Moreover, it aims to advance the global action agenda in Brazil, while contributing with reflections that can serve as a reference for the Latin American region.

### Antibiotic Prescription and Antimicrobial Resistance

Antibiotic prescription has been the subject of increasing monitoring in hospital settings in the last decade, even though great part of the problem relies on PHC. Studies point to several factors associated with the prescription of antimicrobials: knowledge about AMR, critical clinical situations, risk perceptions and patients’ expectations, as well as professionals’ behaviors, values, and ideologies^(4)^. Furthermore, the functioning of health services, the communication between different levels of health care, and the influence, or lack of clinical recommendations and evidence-based clinical protocols, particularly in PHC, pose challenges for the implementation of appropriate interventions at this level of health care. 

Antibiotic prescription has been widely used as a preventive strategy in veterinary medicine and zootechnics. Antibiotics are offered on a large scale to promote the growth and development of animals for human food consumption, although little attention is paid to the impacts these practices have on the development of AMR^(5)^.

However, the purchase, sale, handling and disposal of antibiotics in pharmacies and pet supply stores continue to be addressed only superficially addressed and in a fragmented way^(6)^. Thus, AMR tends to be analyzed, above all, in relation to human health or the doctor-patient relationship, despite the fact that AMR extends into the web of interactions among humans, animals, and the environment, including the health systems themselves and the provision of networks of basic sanitation^(5,7)^. There is little scientific production on the ­relationships among humans, animals, and food consumption, especially in the Brazilian context.

Communication and negotiation between antibiotic ­prescribers and users, as well as the challenges and facilities that professionals find in their clinical practices, cooperate to make the intrinsic dynamics of antibiotic prescription even more complex. For instance, in Brazil, antibiotics were included in the so-called *kit-Covid*, an arrangement of drugs considered “prophylactic”, the use of which was encouraged by the Federal Government to be prescribed and used with the slogan of “early treatment”. As a result, azithromycin, an antibiotic already used in health services, had greater prescription rates and sales throughout 2020 in the country as one of the components of the *kit-Covid* ^(8)^. 

### Antibiotic Use Practices from the Point of View of Health Services Users

In Brazil, the analysis of access conditions to the services of the Brazilian Public Health System (SUS), especially through the teams of the Family Health Strategy (FHS), contributes to the knowledge of housing spaces and the understanding of the factors conditioning demand and the use of antibiotics. It also reveals how the interactions between users and health professionals are important to guide informed attitudes towards the consumption of these medications. The family unit, the community, the relationships between humans and animals, and the sanitary conditions in which they live are aspects for understanding the problem.

Thus, the centrality of the individual inserted in the environment in which they live is strategic, with a focus on ­mapping attitudes and knowledge regarding the use of antibiotics and AMR. Characteristics such as gender, generation, income, and housing are understood as prevalent factors for the different forms of antibiotic use. The individuals’ lack of knowledge about the complications caused by the use of these medications without a medical prescription and through self-medication^(6)^ is identified. The impact of disposing of antibiotics in household waste, sinks, and toilets, as well as its influence on the selective pressure of resistant agents, is not completely known, indicating the relevance of investigating the conditions of use, storage, and disposal of antibiotics among health system users. All these aspects are an urgent issue in the Brazilian context, given the high rate of prescription and consumption of these drugs since the beginning of the COVID-19 pandemic in the country^(8)^.

Such elements demand a comprehensive understanding of the environment, as the context in which individuals and groups are inserted, with the notion of the environment being that of a shared space where networks encompassing individuals and groups, as well as the relationships they establish among themselves, are built. In this regard, the integrated approach to the environment and health, both human and animal, advocated by the One Health, is pertinent. Therefore, dimensions of ­cohabitation and interaction between owners and animals are visible, as both domestic animals and humans can develop and transmit multidrug resistant microorganisms. Consequently, veterinary care practices for domestic animals, such as prescribing antibiotics, also affect the development of AMR.

### The Response to Amr in Brazil: Building a Political Agenda

Although specific AMR-related actions have been developed in previous years, the formalization of a national agenda was only materialized in 2018, with the publication of the National Action Plan for the Prevention and Control of Antimicrobial Resistance in the Scope of One Health (PAN-BR)^(9)^. The PAN-BR was coordinated by the Ministry of Health and included the participation of the Brazilian Health Regulatory Agency (Anvisa), the Ministry of Agriculture, Livestock, and Supply (Mapa), and the Ministry of the Environment (MMA), among others. The PAN-BR both meets an internal need to build a more integrated and intersectoral political agenda, as well as responds to a call from the multilateral agenda to recognize the problem of AMR as a priority issue. This initiative was led by the WHO in partnership with the Food and Agriculture Organization of the United Nations (FAO) and the World Organization for Animal Health (OIE).

The formulation of the PAN-BR represents a step forward in recognizing the need for a multidisciplinary and multisectoral approach to AMR. Its implementation demands articulated relationships among different levels of governance, public and private agencies, industries, as well as the society in general. This implies different priorities and possible conflicting ­interests. Among the main challenges for its implementation, the following are highlighted: the institution of a sustainable policy; the decentralized execution of control, prevention, and ­monitoring activities; and investment in research, development, and innovation for the production of antimicrobials, diagnostic methods, and vaccines through universal and equitable access^(10)^. The creation of mechanisms that allow dialogue between health and epidemiological surveillance, with data sharing, to allow the search for causal links related to AMR from the perspective of One Health is required. 

The context of the global SARS-CoV-2 pandemic highlighted important elements for the AMR agenda, including the urgency of investment in infection prevention and control programs, and the need for comprehensive risk communication strategies, as well as the dangers arising from technological dependence on health supplies for the guarantee of medicines, including antibiotics. The lessons learned can serve as a reference for planning AMR-related interventions in the post-pandemic scenario and for preventing future crisis.

Research on the political agenda of AMR in Brazil implies understanding the frameworks in which AMR is inserted, as well as how the underlying processes of dispute, negotiation, consensus and dissent are constructed based on the perspectives of the main interested actors. Understanding the extent to which policy responses such as the PAN-BR incorporate views and demands from health service users and professionals who work in the prescription and dispensing of antibiotics is essential to promote adequate and sustainable interventions.

## Financial support

Danida – Ministry of Foreign Affairs of Denmark.
